# Supplementary material for: A unified pipeline for FISH spatial transcriptomics
Source: Cell Genom. 2023 Aug 21;3(9):100384. doi: 10.1016/j.xgen.2023.100384 (PMC10504669; doi:10.1016/j.xgen.2023.100384)
Supplement: Document S1. Figures S1–S8 [file mmc1.pdf]

Cell Genomics, Volume *3*

Supplemental information

A unified pipeline for FISH spatial transcriptomics

Cecilia Cisar, Nicholas Keener, Mathew Ruffalo, and Benedict Paten

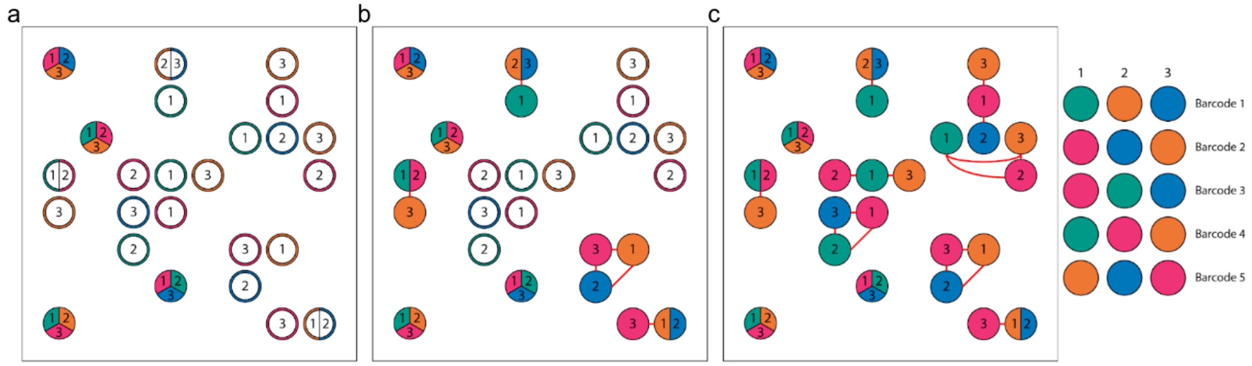

Figure S1: **Schematic representation of the results of different seqFISH decoding methods, related to Figure 1.**

Each circle represents a spot in a theoretical seqFISH experiment with the number denoting the imaging round and the color corresponding to the imaging channel. Split spots show spatially co-occurring spots, filled in spots are part of a decodable barcode for the decoding method shown in that panel and red lines connect spots of the same decodable barcode that do not spatially co-occur. The codebook is shown on the far right. Decoding methods shown are **A**) starfish ExactMatch decoder, **B**) starfish NearestNeighbor decoder, and **C**) the custom CheckAll decoder.

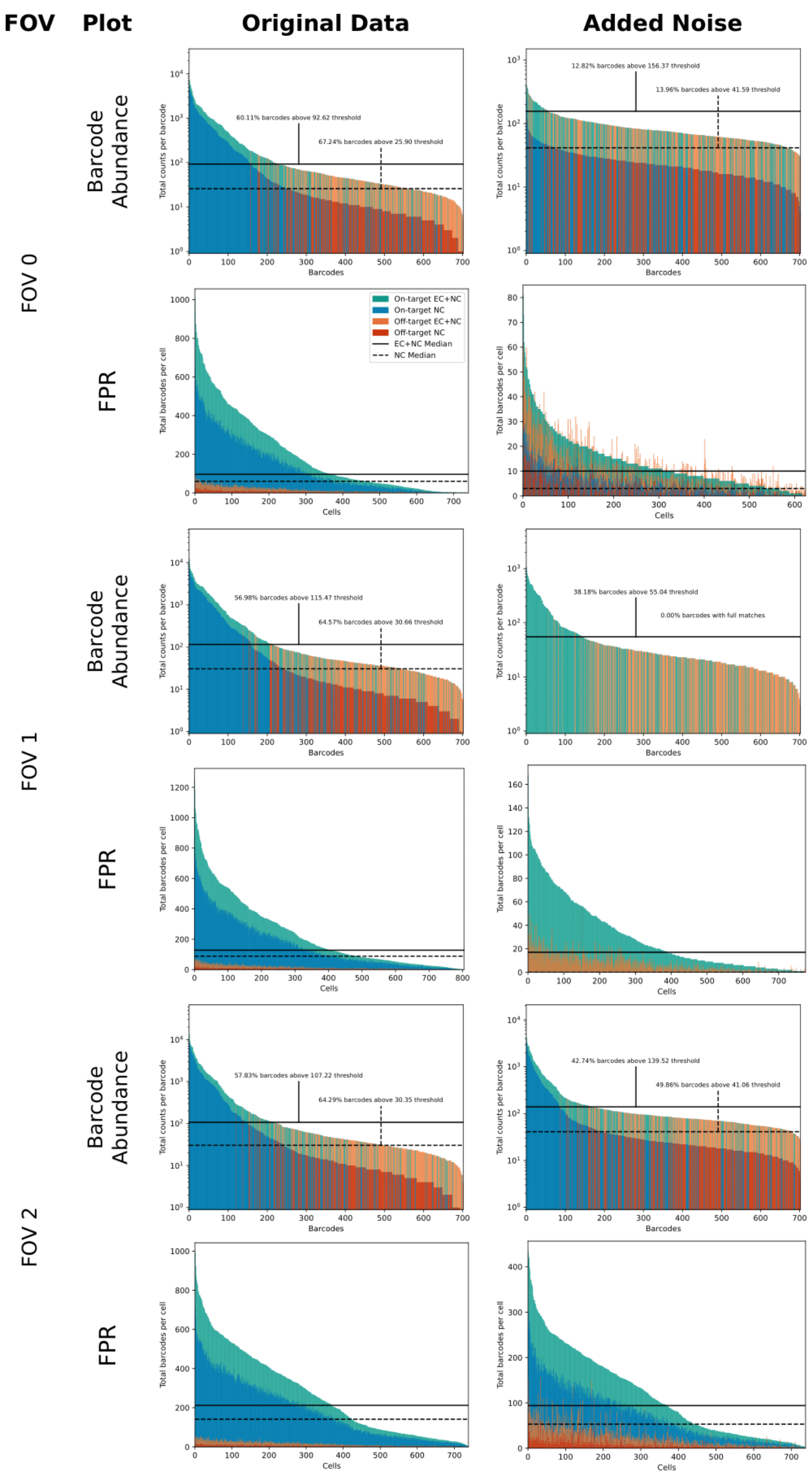

Figure S2: **Comparison of selected QC metrics on data before and after simulated noise, related to Figure 2.**

Original data taken directly from pipeline output of seqFISH data, “Added Noise” results were obtained by adding noise to the seqFISH images (**Methods**) and then running the same pipeline. EC = error-corrected, NC = non-corrected.

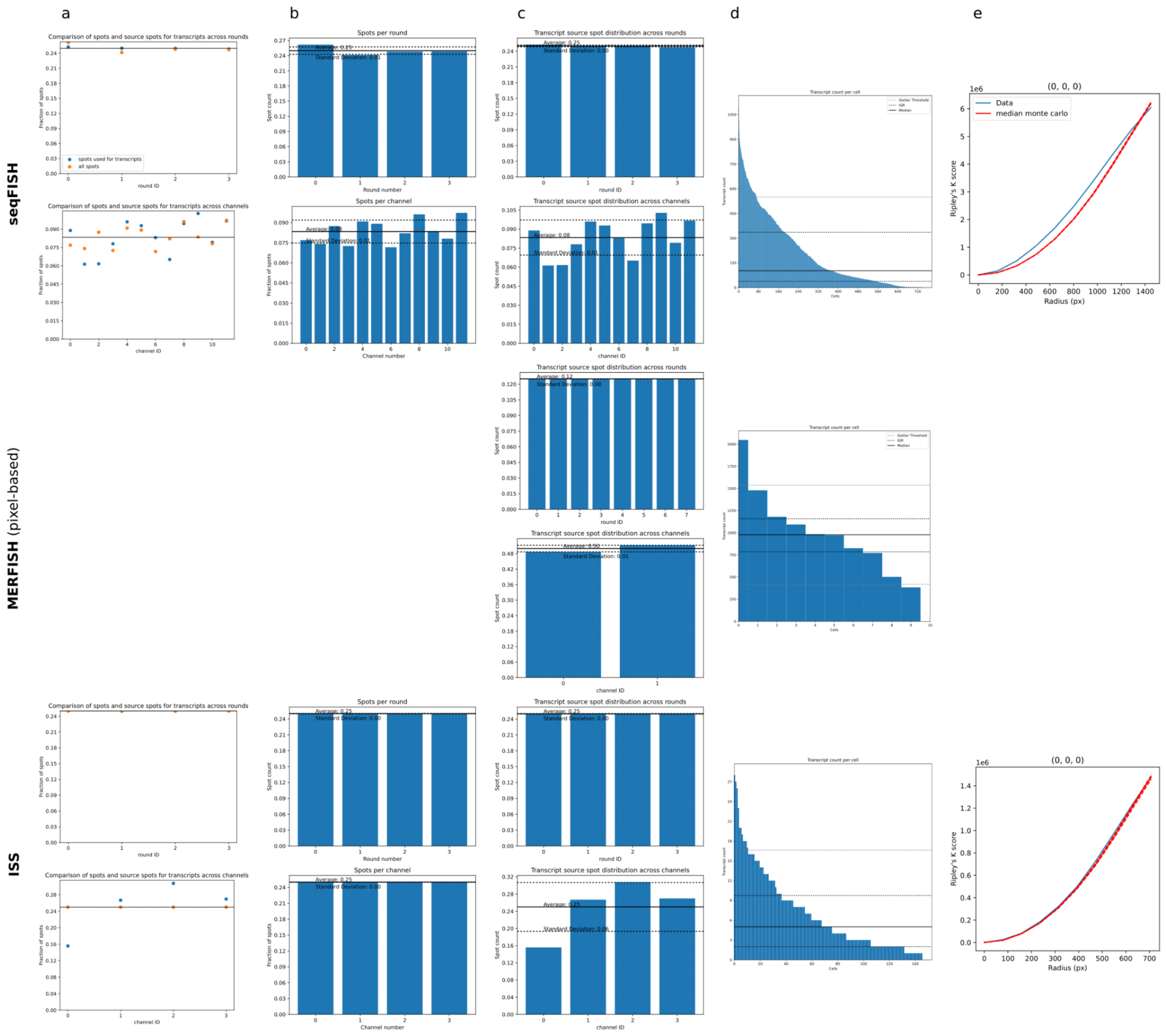

Figure S3: Additional QC Metrics for each dataset, related to STAR Methods. Data is from the first FOV of each experiment, all plots are taken directly from pipeline output. Note that because MERFISH uses a pixel-based method, metrics that use spot data do not apply. **A**) Comparison of source spots for transcripts and all spots detected *(top)* across imaging channels and *(bottom)* across imaging rounds. **B**) Spot distribution *(top)* across rounds and *(bottom)* across channels. **C**) Transcript source spot distribution *(top)* across rounds and *(bottom)* across channels. **D**) Transcript count per cell. Outlier threshold is calculated as Median +/- 1.5*IQR **E**) Ripley's k score at increasing radius for spots, shown for the first z-slice of the first round and first channel. Solid line is median score under the null hypothesis (complete randomness) calculated by monte carlo method, dashed lines are 95% confidence interval calculated by monte carlo.

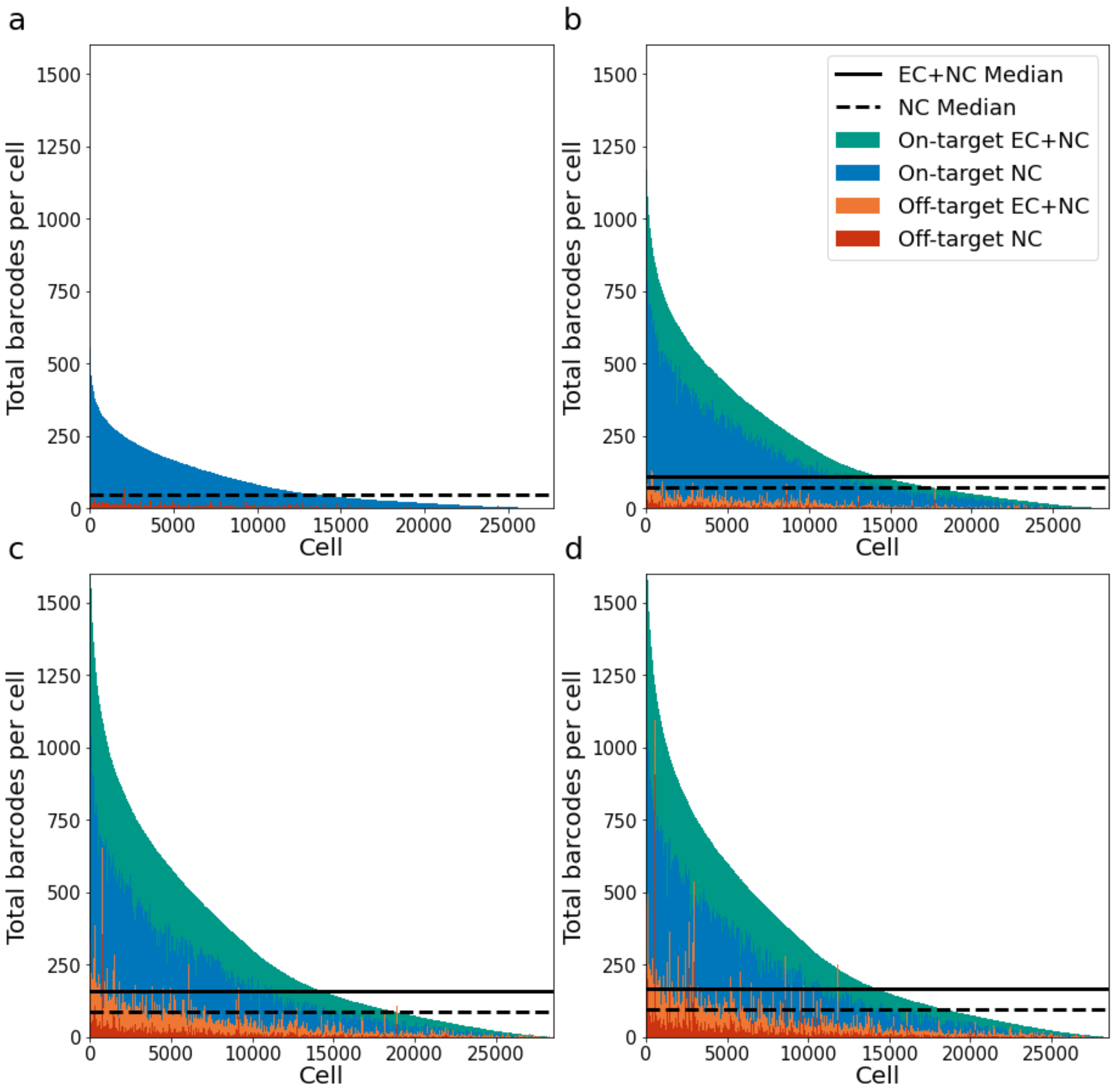

Figure S4: **False positive metric results for starfish and CheckAll decoders, related to Table 1.**

Comparison of performance for **A**) starfish NearestNeighbor decoder **B**) CheckAll decoder (high accuracy mode), **C**) CheckAll decoder (medium accuracy mode), and **D**) CheckAll decoder (low accuracy mode). Total counts of each barcode colored by barcode type. Error-corrected counts added to top of the non-corrected counts. EC = error-corrected, NC = non-corrected. Each column shows values for the same cell, ordered by their on-target NC+EC counts.

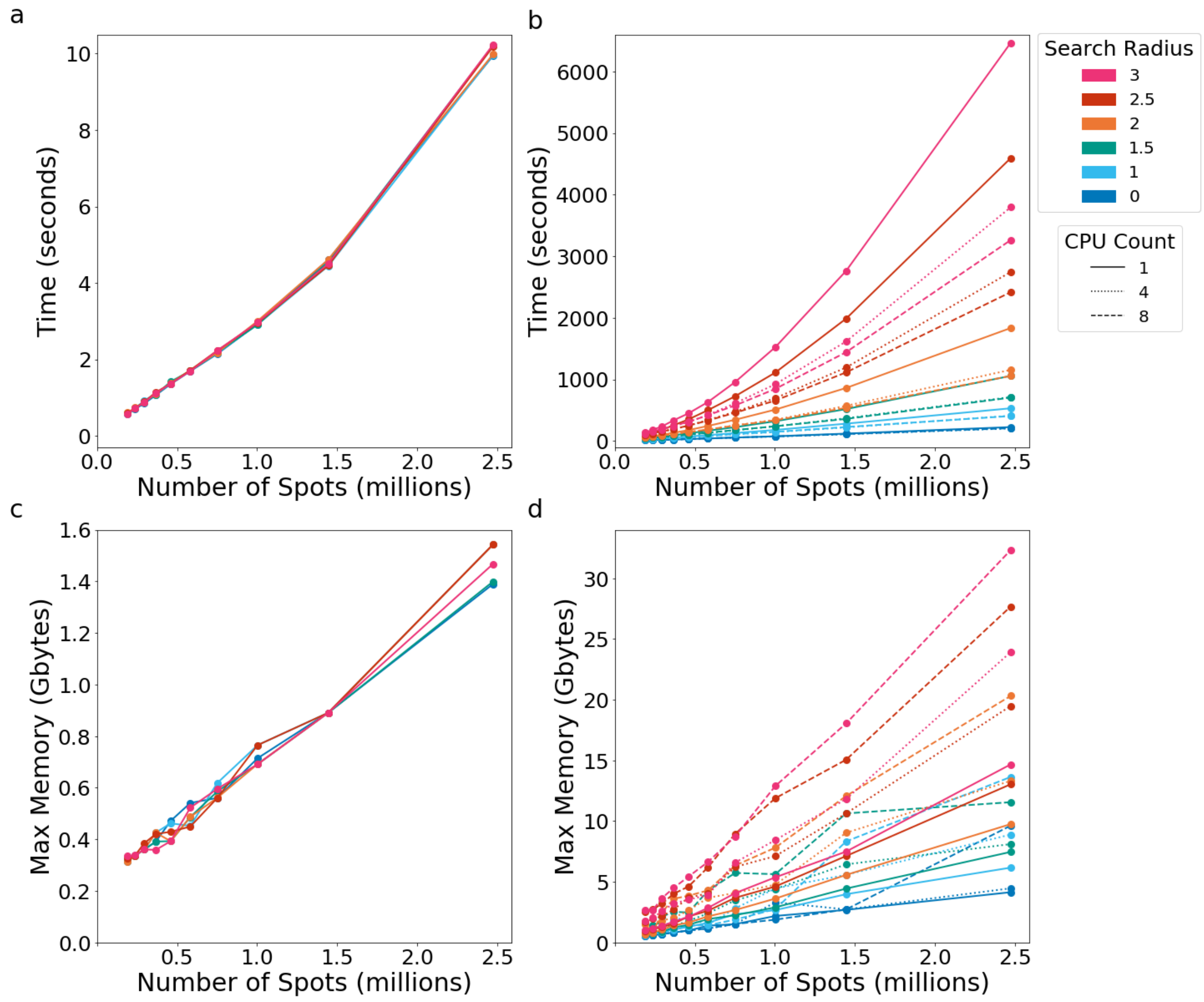

Figure S5: **Run time and memory benchmarks for starfish and CheckAll decoders, related to STAR Methods.**

Comparison of time benchmarks for **A**) starfish NearestNeighbor decoder, **B**) CheckAll decoder and memory benchmarks for **C**) starfish NearestNeighbor decoder, **D**) CheckAll decoder. Results shown for different numbers of input spots, search radii, and CPU's used for multiprocessing (CheckAll decoder only). Run on an AMD Ryzen 9 3900X (3.8 GHz base clock speed).

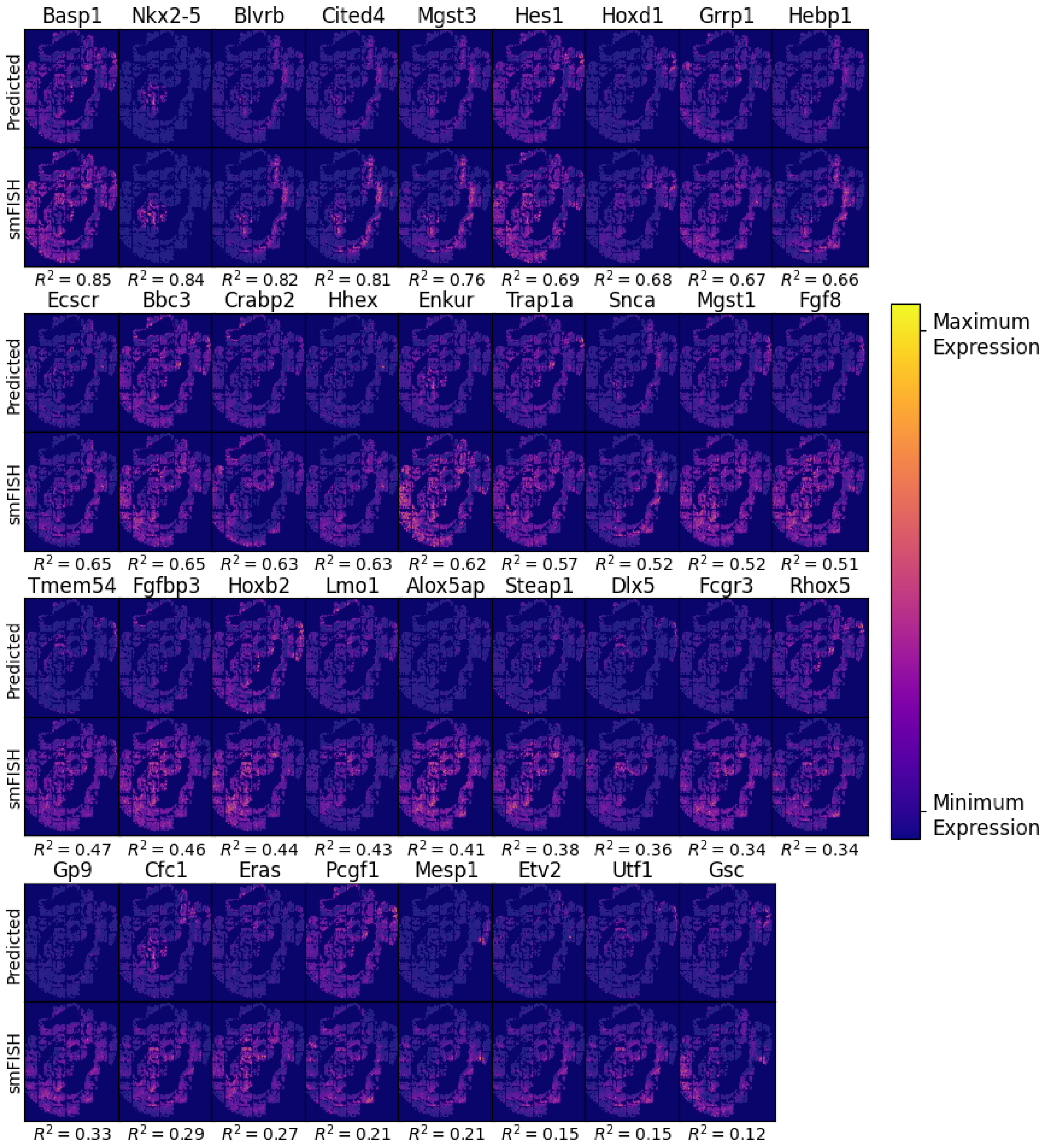

Figure S6: Comparison of predicted and measured expression for all 36 smFISH genes, related to Figure 3.

For each gene, predicted expression by Tangram using seqFISH and scRNA-seq counts is shown on top and the measured expression by smFISH is shown on the bottom. The Pearson correlation of counts across all cells for each gene is printed below each and genes are ordered by the Pearson correlation. The gene ‘Ifng’ was measured by smFISH but is not shown here because it doesn’t appear in the scRNA-seq data used.

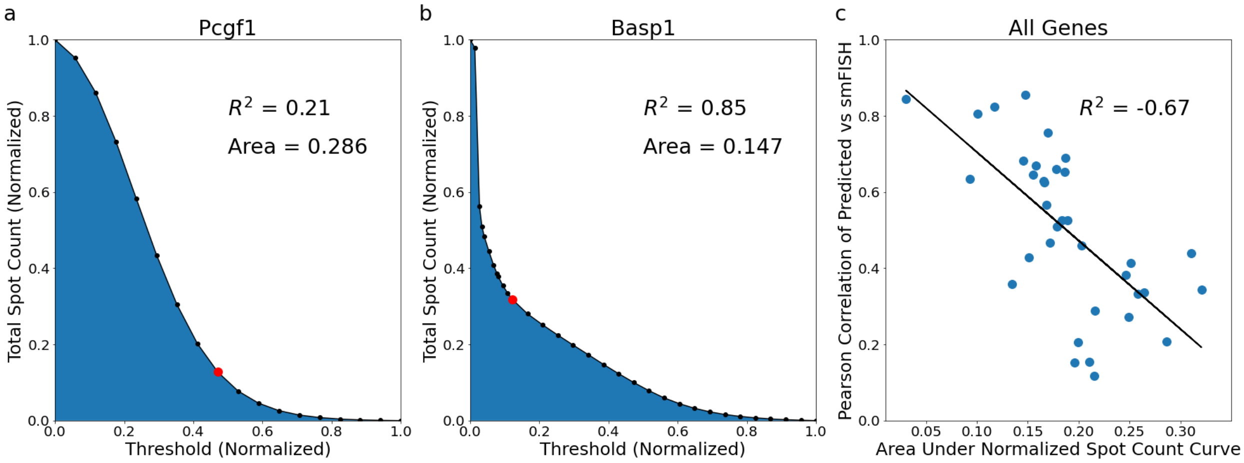

Figure S7: Explanation of poorly performing genes for seqFISH external QC, related to Figure 3.

Normalized total spot vs threshold curves for smFISH images for **A**) Pcgf1 and **B**) Basp1. Printed R^2 is the Pearson correlation between predicted and smFISH counts for that gene while the area is the integral of the normalized total spot vs threshold curve. The red highlighted point indicates the elbow point of the curve that was used as the threshold value to obtain the printed Pearson correlation. **C**) Pearson correlation of predicted and smFISH counts vs the area under the normalized spot count curve for all genes. R^2 printed is the Pearson correlation between the Pearson correlation of predicted and smFISH counts and the area under the normalized spot count curve.

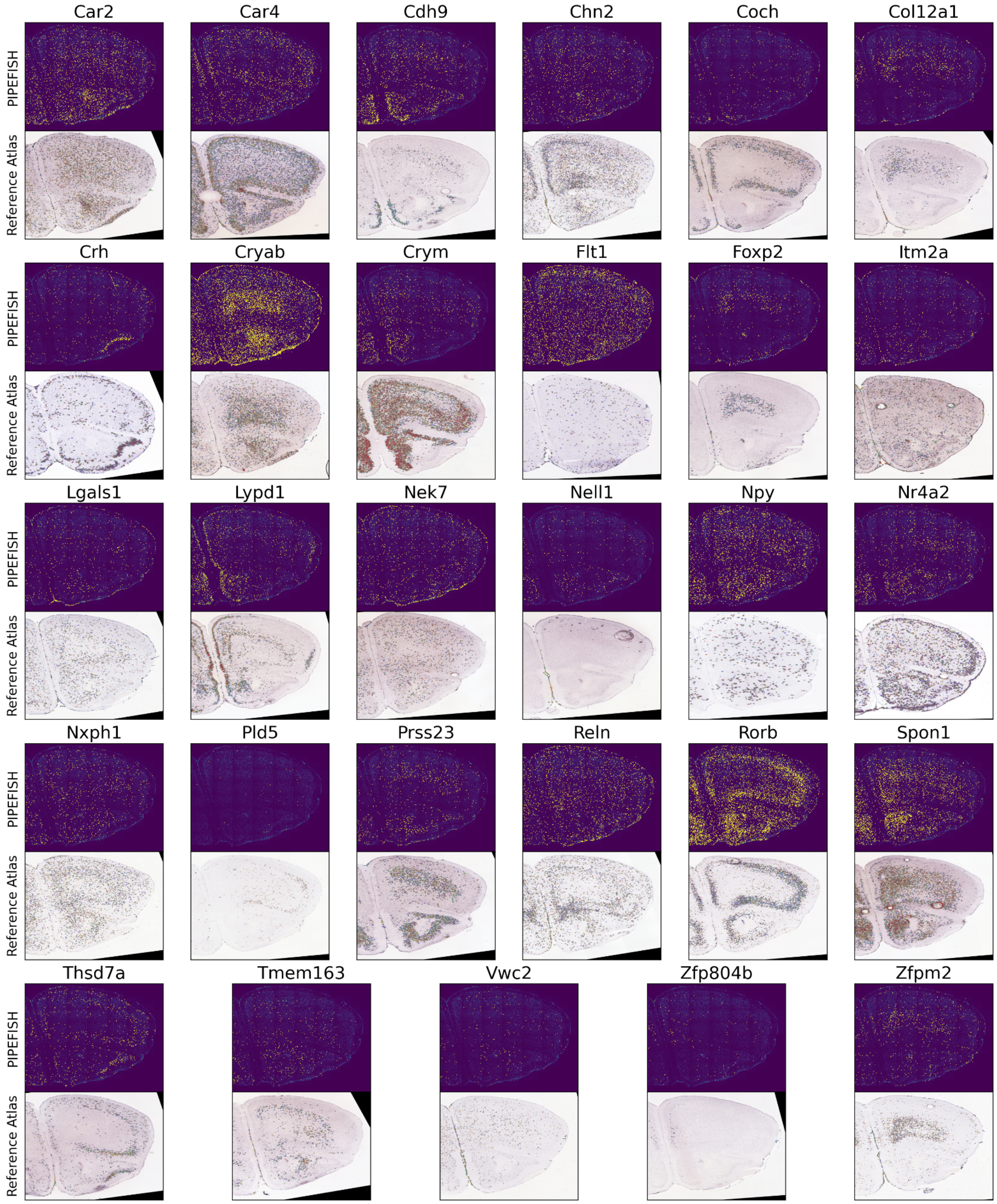

Figure S8: **Comparison of ISS pipeline expression with reference atlas, related to Figure 3.**

All 29 additional genes found in the mouse brain ISS dataset with a coronal section reference in the Mouse Brain Atlas. In the top row: each yellow dot represents a transcript while the image underneath is the DAPI stain of the sample, in the bottom row: blue dots represent low expression while more red dots represent higher expression (no color map provided by Allen Brain Atlas) while the image underneath is the Nissl stain of the sample.
